# Linkja: Open Source Privacy-Preserving Record Linkage Tool

**DOI:** 10.23889/ijpds.v9i5.2852

**Published:** 2024-09-18

**Authors:** Kruti Doshi, William Trick, Keiki Hinami

**Affiliations:** 1 Medical Research Analytics and Informatics Alliance; 2 Cook County Health

## Objectives

To introduce Linkja, an open-source, certified, Privacy-Preserving Record Linkage (PPRL) tool with the primary objective of facilitating secure and efficient record linkage across diverse datasets, while preserving individual privacy, providing a comprehensive and privacy-centric approach to program assessments.

## Approach

Linkja has been used to evaluate Chicago’s Flexible Housing Pool program by securely linking various datasets, including Homeless Management Information Systems (HMIS), Electronic Health Records, Jail records, Medical Examiner reports, and Public Health data. Data contributors used separate honest brokers for record linkage, and generation of salt and cryptography keys.

## Results

Linkja enabled this multi-sector linkage by facilitating legal and data sharing agreements, avoided licensing fees and procurement processes for all parties, and provided a de-identified for analysis. The insights gleaned from the analysis motivated the FHP program to plan a follow-up evaluation that will add emergency serviced records from a separate City of Chicago department. The successful linkage provided a nuanced understanding of the association between stable housing and reduced healthcare utilization.

## Conclusions

Linkja's cryptographic certification ensures the highest standards of security during data linkage, safeguarding sensitive information. The matching rules, validated on hard-to-match populations, prove essential in maintaining accuracy and reliability in the linkage process.

## Implications

The open-source nature of Linkja promotes transparency and collaboration in the development and enhancement of privacy-preserving record linkage techniques. This tool has broad implications for sectors dealing with sensitive data, fostering responsible data-sharing practices, and enabling secure collaborative research initiatives without compromising individual privacy.

